# Diabetogenically beneficial gut microbiota alterations in third trimester of pregnancy

**DOI:** 10.1530/RAF-20-0034

**Published:** 2021-01-05

**Authors:** Emmanuel Amabebe, Dilly O Anumba

**Affiliations:** 1 Department of Oncology and Metabolism, University of Sheffield, Sheffield, UK

**Keywords:** pregnancy, gut, microbiota, diabetes, metabolic syndrome, inflammation

## Abstract

Altered gut microbiota (dysbiosis), inflammation and weight gain are pivotal to the success of normal pregnancy. These are features of metabolic syndrome that ordinarily increase the risk of type 2 diabetes in non-pregnant individuals. Though gut microbiota influences host energy metabolism and homeostasis, the outcome (healthy or unhealthy) varies depending on pregnancy status. In a healthy pregnancy, the gut microbiota is altered to promote metabolic and immunological changes beneficial to the mother and foetus but could connote a disease state in non-pregnant individuals. During the later stages of gestation, metabolic syndrome-like features, that is, obesity-related gut dysbiotic microbiota, increased insulin resistance, and elevated pro-inflammatory cytokines, promote energy storage in adipose tissue for rapid foetal growth and development, and in preparation for energy-consuming processes such as parturition and lactation. The origin of this gestation-associated host–microbial interaction is still elusive. Therefore, this review critically examined the host–microbial interactions in the gastrointestinal tract of pregnant women at late gestation (third trimester) that shift host metabolism in favour of a diabetogenic or metabolic syndrome-like phenotype. Whether the diabetogenic effects of such interactions are indeed beneficial to both mother and foetus was also discussed with plausible mechanistic pathways and associations highlighted.

**Lay summary:**

In non-pregnant women, increased blood glucose, fat accumulation, and prolonged immune response lead to obesity and diabetes. However, during the later stages of pregnancy, the changes in the body’s metabolism described previously do not lead to disease, instead pregnancy facilitates the storage of sufficient energy in fat cells for rapid growth and development of the foetus. The excess energy stores also prepares the mother for labour and breastfeeding. This review examines the role of the normal bacteria in the digestive tract in this beneficial energy accumulation and transfer between the mother and foetus without leading to obesity, diabetes and hypertension in pregnancy.

## Introduction

Metabolic syndrome (MetS) is characterised by a spectrum of clinical findings (phenotypes) including hyperglycaemia, insulin resistance, increased adiposity, and chronic subclinical inflammation, that aggravate an individual’s risk of type 2 diabetes mellitus (T2DM) (Koren *et al.* 2012). Though there are several definitions of MetS, a unified international definition incorporating the definition by the National Cholesterol Education Program Adult Treatment Panel III (NCEP/ATPIII) has been recommended. The definition also proposed the use of population-specific waist circumference thresholds to determine obesity (Samson & Garber 2014). The harmonised definition of MetS issued in 2009 is presented in Table 1.

**Table 1:** Harmonised definition of metabolic syndrome.

	Clinical feature
Criteria/no. of risk factors	≥3 of the risk factors below
Obesity	WC: population-specific values*
Hyperglycaemia	FPG ≥5.6 mmol/L (100 mg/dL) or treated
Hypertension	SBP ≥130 mmHg DBP ≥85 mmHg or treated
Dyslipidaemia	HDL-C: <1.0 mmol/L (40 mg/dL) (M), <1.3 mmol/L (50 mg/dL) (F) TG: ≥1.7 mmol/L (150 mg/dL) or treated

This definition is a joint statement issued in 2009 by the International Diabetes Federation, American Heart Association/National Heart, Lung and Blood Institute, the World Heart Federation, the International Atherosclerosis Society, and the International Association for the Study of Obesity.

*Population-specific waist circumference values can be found in Samson and Garber (2014).

DBP, diastolic blood pressure; F, female; FPG, fasting plasma glucose; HDL-C, high-density lipoprotein-cholesterol; M, male; SBP, systolic blood pressure; TG, triglyceride; WC, waist circumference.

The over 100 trillion resident bacteria in the human gastrointestinal tract (gut microbiota) are crucial to human metabolism, immunity and overall health (Edwards *et al.* 2017, Amabebe *et al.* 2020). Due to its immense contribution to the maintenance of homeostasis, the gut microbiota has been described as a microbial organ (Edwards *et al.* 2017, Amabebe *et al.* 2020). The constituent microorganisms, which are acquired at birth, evolve through a mutualistic relationship with the host (Liu *et al.* 2017). This microbial evolution continues throughout life influenced by several factors including genetic factors, mode of delivery, maternal and infant dietary habit, age, environment and pregnancy (Saad *et al.* 2016, Edwards *et al.* 2017, Ponzo *et al.* 2019).

Pregnancy induces immunological and metabolic changes manifested as immune tolerance that allow implantation and placentation; and insulin resistance to support growth of the foetus (Crusell *et al.* 2018). Because the later stage (especially third trimester) of pregnancy is characterised by MetS-like features, that is, maternal hyperglycaemia, increased adiposity, high levels of circulating proinflammatory chemocytokines and insulin resistance; it has been described as a diabetogenic state (Mor & Cardenas 2010). These physiological changes can even lead to gestational diabetes in high-risk women (Crusell *et al.* 2018).

Considering the interrelationship between pregnancy, obesity and diabetes through their common features, that is, low-grade systemic inflammation, altered gut microbial composition, insulin resistance and hyperglycaemia, the evaluation of these features and their association especially in relation to maternal and foetal health is of great importance. Interestingly, the MetS-like phenotype observed in later gestation is not overtly harmful to the mother and foetus in most cases. In fact, it has been described as beneficial and necessary for a successful parturition and postpartum health (Collado *et al.* 2008, Santacruz *et al.* 2010, Koren *et al.* 2012, Edwards *et al.* 2017, Liu *et al.* 2017). However, the mechanistic link between these diabetogenic phenotypes and periparturition health and safety of the fetomaternal unit without leading to gestational diabetes or other complications, is still unresolved and deserves more attention. Therefore, this review critically examined the host–microbial interactions in the gastrointestinal tract of pregnant women at late gestation (third trimester) that shift host metabolism in favour of a diabetogenic or metabolic syndrome-like phenotype. Whether the diabetogenic effects of these interactions are indeed beneficial to both mother and foetus was also discussed with plausible mechanistic pathways and associations highlighted.

## Pregnancy-induced microbial changes

The endocrine and physical changes that accompany pregnancy trigger an array of anatomical, physiological and biochemical alterations that affect every organ of the body (Prince *et al.* 2014). The microbiome of different body sites including the vagina and gut which are in a continuous crosstalk (Amabebe & Anumba 2020) also exhibit immunological and metabolic changes associated with the progression of normal, healthy gestation. At the onset of pregnancy, the immune system represses itself to allow and support implantation of the foetus, and restrengthens towards mid and later stages of pregnancy to prevent invasion by pathogens (Edwards *et al.* 2017). This ‘rewiring of immune system’ throughout pregnancy induces a low-grade inflammation at the mucosal surfaces of gut, oral cavity, vagina and placenta. This leads to structural and compositional changes in the microbiota of these body sites (Edwards *et al.* 2017).

The vaginal microbiota at late gestation is similar to that of a non-pregnant (non-menstruating and bacterial vaginosis, BV-negative) state characterised by a decrease in α-diversity (species richness – within individual diversity or amount of different species detected in the vaginal sample), and a corresponding increase in *Lactobacillus* spp. (Kaur *et al.* 2020). This resemblance is believed to trigger the changes that are associated with parturition. As a prerequisite for a successful pregnancy, the vaginal microbiome shifts in favour of *Lactobacillus* spp. dominance, becomes more stable and less diverse. Of course, this is not without the support of rising oestrogen, increased vaginal epithelial glycogen accumulation, and increased acidity (Amabebe & Anumba 2018, 2020, Kaur *et al.* 2020).

Both *crispatus* and *jensenii* increase as part of the vaginal microbiomial stability associated with gestation. These species metabolise glycogen hydrolysates to produce lactic acid to propagate their growth and replication. This is facilitated by high levels of oestrogen that stimulates glycogen accumulation in vaginal epithelial cells and epithelial-derived α-amylase that depolymerises the α-glucan molecules (Amabebe & Anumba 2018, Tester & Al-Ghazzewi 2018). In addition to the lactic acid, the lactobacilli produce hydrogen peroxide and bacteriocins that inhibit the growth of BV-associated pathogens (Amabebe & Anumba 2018, Tester & Al-Ghazzewi 2018), thereby preventing infection associated spontaneous preterm birth (Prince *et al.* 2014).

The gestation-associated changes in the vaginal microbiota also have postnatal benefits to the offspring. *Lactobacillus johnsonii* is primarily found in the upper gut where it sustains its survival and persistence by catalysing the hydrolysis (i.e. release of taurine and glycine) and uptake of bile through its bile salt hydrolase and transporters (Pridmore *et al.* 2004). It inhibits the growth and survival of other lactobacilli and enterococci in the gut through the production of bacteriocin – Lactacin F (Abee *et al.* 1994). These properties aid postnatal digestion of breast milk by neonates inoculated with vaginal *L. johnsonii* at birth (Prince *et al.* 2014).

## Non-pregnant gut microbiota

*Firmicutes* and *Bacteroides*, which constitute >95% of the bacterial population, dominate the physiologic gut microbiota in the non-pregnant state. Other bacteria phyla/divisions present in small proportions include *Proteobacteria*, *Fusobacteria*, *Actinobacteria* and *Verrucomicrobia* (Amabebe & Anumba 2020, Amabebe *et al.* 2020). These microorganisms ferment non-digestible dietary fibres to produce metabolites (short chain fatty acids, SCFAs) that maintain intestinal barrier integrity and prevent leakage of bacteria and bacterial products such as lipopolysaccharide (LPS) in to systemic circulation (Amabebe & Anumba 2020, Amabebe *et al.* 2020). The microbiota is distributed throughout the gut varying according to region and determined by pH, nutrient, and oxygen availability (Saad *et al.* 2016). Overall, the gut microbes influence energy utilisation, hormonal, immunologic and metabolic health of the host. Alterations in the gut microbial composition is linked to metabolic and immunologic conditions including obesity, T2DM, inflammatory bowel disease (IBD), irritable bowel syndrome, allergies, pre-eclampsia, gestational diabetes, etc. Detailed review of the role of gut microbiota in health and disease can be found in the following reports (Canfora *et al.* 2015, Saad *et al.* 2016, Chambers *et al.* 2018, Tungland 2018, Lazar *et al.* 2019, Parada Venegas *et al.* 2019, Amabebe & Anumba 2020, Amabebe *et al.* 2020).

## Gut microbiota changes in late gestation

At the beginning of pregnancy, the gut microbiota profile resembles that of a healthy non-pregnant woman characterised by predominance of *Firmicutes* (particularly *Clostridiales*, *Faecalibacterium prausnitzii*) over *Bacteroidetes* (Walters *et al.* 2014). Subsequently, gut microbiota is greatly altered over the course of pregnancy. Maternal gut function and microbiota are altered by the unique physio-biochemical, inflammatory and immune responses that occur as pregnancy advances. These alterations permit changes in maternal metabolism and weight required to maintain pregnancy and survival of the foetus after delivery (Koren *et al.* 2012, Edwards *et al.* 2017).

**Figure 1:**
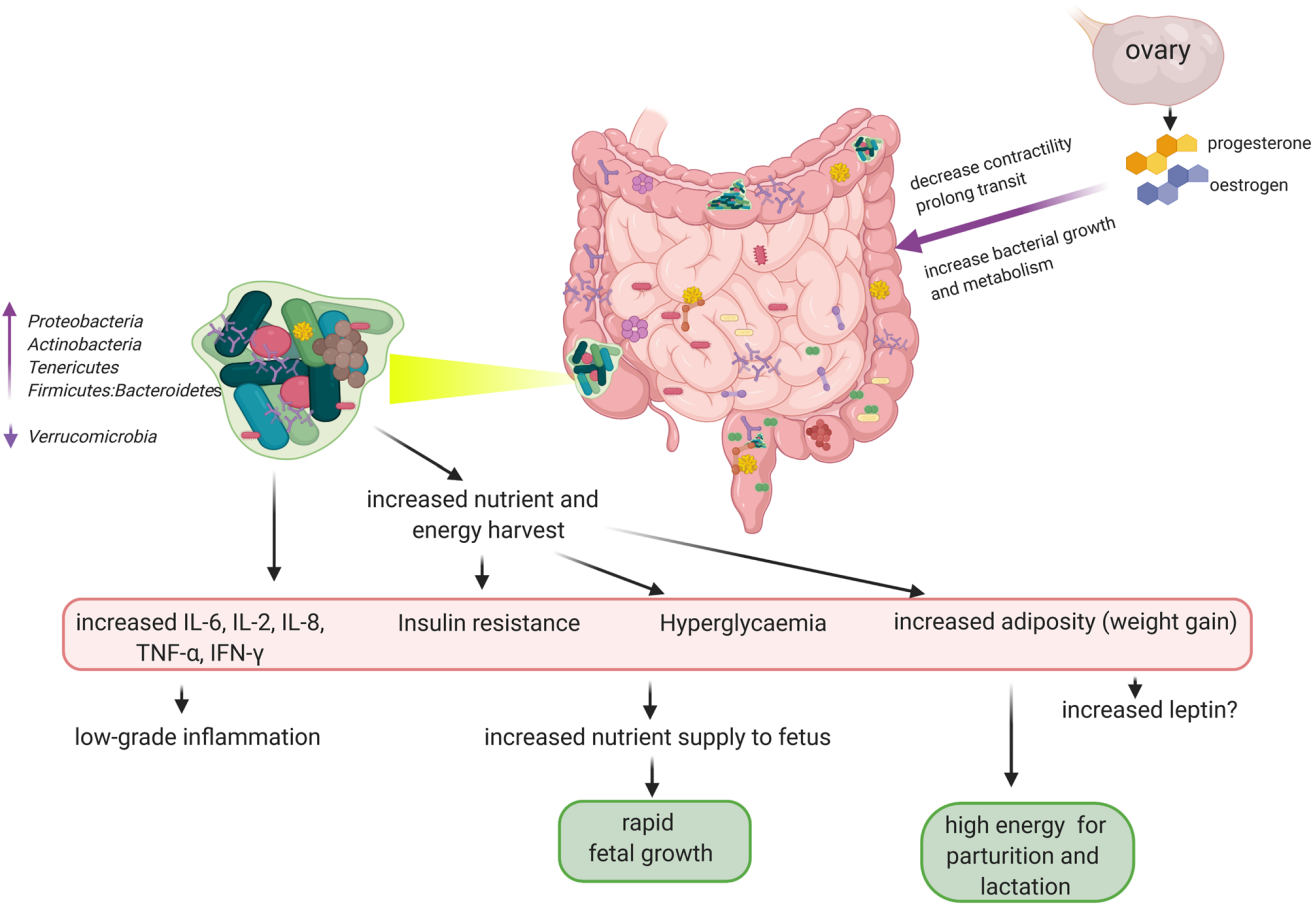
Third trimester-associated diabetogenically beneficial gut microbiota. As pregnancy advances, hormonal and immunological changes increase nutrient and energy harvest from the gut. These changes are induced by increased oestrogen and progesterone that inhibit gastrointestinal contractility and prolong transit providing a suitable environment (substrates) for energy-harvesting microbes. The consequent dysbiosis triggers a low-grade inflammatory state propagated by pro-inflammatory chemocytokines leading to insulin resistance and hyperglycaemia. This diabetogenic phenotype preferentially supplies abundant nutrients to the maturing foetus and prepares the mother for energy-demanding processes of parturition and lactation through the accumulation of fat and weight gain. There are also possibilities of adiposity-induced increased leptin and perhaps leptin resistance as seen in obesity. Though these are typical features of metabolic syndrome, they are not usually detrimental to maternal and fetal health in the later stage of gestation. *Created with Biorender.com*.

In a European cohort, the gut microbiota species richness or within individual α-diversity (Ponzo *et al.* 2019) decreased from the first to third trimester with overall increase in *Proteobacteria* (*Enterobacteriaceae*, *Escherichia coli*) and *Actinobacteria* (*Propionibacterium*) (Fig. 1); whereas there was an increase in inter-subject/sample/site β-diversity. A reduction in the health-promoting, butyrogenic and anti-inflammatory *Faecalibacterium prausnitzii* is also observed by the third trimester (Koren *et al.* 2012, Garcia-Mantrana & Collado 2016, Nuriel-Ohayon *et al.* 2016). This is similar to the microbial composition observed in non-pregnant adults with MetS (Koren *et al.* 2012). *F. prausnitzii* abundance is inversely related with low-grade inflammation, plasma glucose and diabetes; with a capacity to predict the risk of T2DM (Ferrocino *et al.* 2018). These microbial alterations permit a chronic low-grade inflammation at the mucosal surfaces of the gut; and may be influenced by diet, antibiotic therapy, pre-pregnancy BMI and gestational diabetes. However, such microbial alterations are usually not related to these factors (Koren *et al.* 2012, Gohir *et al.* 2015, Mesa *et al.* 2020).

Furthermore, the microbial changes and adaptations may be gene and/or environment-dependent as other phyla were either decreased (i.e. *Verrucomicrobia*) or increased (i.e. *Tenericutes*) (Fig. 1) in the third trimester in a pregnant Southern Chinese cohort. Moreover, *Bacteroidetes* (acetate and propionate producers) were the most abundant bacteria in these women, while *Firmicutes* (major butyrate producers) reduced relatively (Liu *et al.* 2017). Butyrate has shown more anti-inflammatory activities than acetate and propionate, hence, frequently used in the treatment of inflammatory bowel disease (IBD, ulcerative colitis and Crohn’s disease) and colorectal cancers (Cox *et al.* 2009, Mirmonsef *et al.* 2011, 2012, Koh *et al.* 2016, Rivière *et al.* 2016, Parada Venegas *et al.* 2019). Alas, the intestinal levels of SCFAs were not determined in this Chinese cohort.

Animal models have demonstrated *Verrucomicrobia* is associated with poor digestion and absorption of nutrients (mice) (Preidis *et al.* 2015), while increased *Tenericutes* enhance nutrient digestion and absorption (i.e. increased energy harvest) in pigs (Niu *et al.* 2015). The increase in *Tenericutes* and decrease in *Verrucomicrobia*, and the corresponding enhanced energy harvest observed in late gestation of some Chinese women may be a similar adaptation to the high energy demands required for foetal growth and maturation, parturition and lactation (Fig. 1) (Liu *et al.* 2017). The strengthened digestive and absorptive capacity could be an adaptive mechanism required for the higher energy and metabolic processes in late pregnancy (Liu *et al.* 2017).

**Figure 2:**
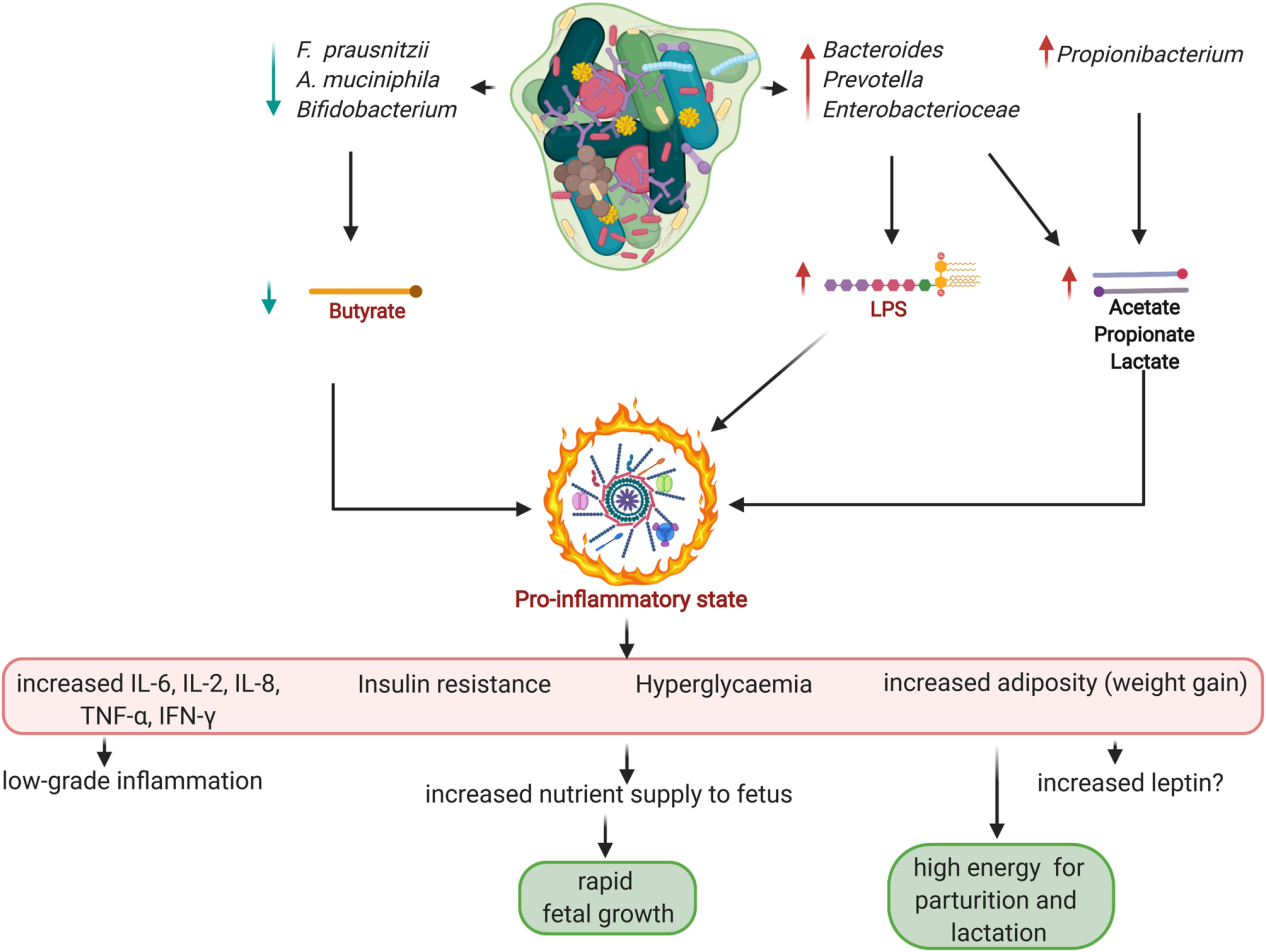
Gut microbial-metabolite profile in third trimester of normal pregnancy. The low-grade pro-inflammatory state that leads to glucose dysmetabolism and insulin resistance albeit non-injurious to mother and foetus, may be a constellation of high lipopolysaccharide (LPS) from the predominating Gram-negative species – *Bacteroides*, *Prevotella* and *Escherichia coli*; and low butyrate levels due to a decline in butyrogenic species – *Faecalibacterium*, *Akkermansia*, *Bifidobacterium*. High acetate, propionate and lactate produced by the Gram-negative species in collaboration with *Propionibaterium* are unable to inhibit the pro-inflammatory state. Hence, the declining butyrate level may be the determinant factor. *Created with Biorender.com*.

Additionally, the low abundance of probiotic *Firmicutes* such as *Coprococcus catus* (Liu *et al.* 2017) and *F. prausnitzii* (which lack LPS) (Koren *et al.* 2012, Garcia-Mantrana & Collado 2016, Nuriel-Ohayon *et al.* 2016), in relation to *Bacteroidetes* (that possess high LPS) may lead to elevated LPS production and subsequent inflammation that may promote the MetS-like phenotype at late gestation (Fig. 2) (Liu *et al.* 2017). However, this study (Liu *et al.* 2017) did not indicate the species of the *Bacteroidetes* phyla that may be associated with the proposed elevated LPS.

In the non-pregnant state, gut bacterial LPS can induce a chronic subclinical inflammatory response and obesity, leading to insulin resistance by activation of toll-like receptor (TLR)-4 (Saad *et al.* 2016). It is noteworthy that some genera and species of the *Bacteroidetes* phyla are obesogenic (e.g. *Prevotella* spp.) (Amabebe *et al.* 2020) and diabetogenic (e.g. *fragilis*) (Mancuso *et al.* 2005), while others are anti-obesogenic (e.g. *Bacteroides thetaiotaomicron*) (Kobyliak *et al.* 2016, Amabebe *et al.* 2020). This LPS induced *Bacteroidetes*/*Firmicutes* relationship with diabetogenic phenotype at later gestation requires resolution down to species level for a more comprehensive understanding of the mechanistic pathways.

### Firmicutes: *Bacteroidetes* ratio

Non-pregnant and first trimester pregnant women share similar gut microbial diversity (Santacruz *et al.* 2010). However, as pregnancy advances, the abundance of inflammation-associated gut bacterial species increases in most women (Santacruz *et al.* 2010). Maternal pregestational BMI and weight gain over gestation can also influence the shift in gut microbiota (Collado *et al.* 2008, Santacruz *et al.* 2010, Zacarías *et al.* 2018). Low-grade systemic inflammation, insulin resistance and a dysbiotic gut microbiota are characteristic of increased adiposity as seen in obesity and pregnancy especially the third trimester (Zacarías *et al.* 2018). Similar to the obesity and MetS-related symptoms in non-pregnant state, the ratio of potentially pathogenic (pro-inflammatory) *Firmicutes* to *Bacteroidetes* increases in relation to gestational age especially in obese women. Therefore, at third trimester, there could be more *Firmicutes* than *Bacteroidetes* depending on the mother’s BMI. Elevated levels of potentially pathogenic (pro-inflammatory) *Firmicutes* (e.g. *Clostridium perfringens*) and ultimately higher *Firmicutes*:*Bacteroidetes* ratio was recently observed in overweight and obese pregnant women in the third trimester compared to their normal weight counterparts (Zacarías *et al.* 2018). However, the anti-inflammatory health-promoting *Firmicutes,* that is, *F. prausnitzii* and other butyrate producers of the *Lachnospiraceae* family are decreased in obesity (Chakraborti 2015) and IBD (Rivière *et al.* 2016). The *Firmicutes* are also high energy harvesters, that is, they metabolise non-digestible polysaccharides for example, starch, glycogen, etc., to release energy (Santacruz *et al.* 2010). It was suggested that this MetS-like host–microbial interaction might be importantly beneficial in pregnancy, for energy storage, foetal growth, parturition and lactation. The plausible mechanisms underlying these speculations are; elevated dietary energy extraction efficiency; and disrupted host–microbial interactions that induce metabolic inflammation. Taken together, gestation-associated changes (imbalance) in gut microbiota with corresponding increase in energy harvesting microbes could induce metabolic inflammation (Koren *et al.* 2012).

However, as shown in Fig. 1, it appears the relative abundance of *F. prausnitzii*, an anti-inflammatory *Firmicutes* that constitutes a substantial part of the normal gut microbiota along with other butyrate-producing *Firmicutes* (Lopetuso *et al.* 2013, Chakraborti 2015, Amabebe & Anumba 2020) influences the overall immunometabolic phenotype observed in later gestation regardless of the *Firmicutes*:*Bacteroidetes* ratio. That is, even though the *Firmicutes*:*Bacteroidetes* ratio remains high in the later stages of gestation as seen in obesity, a low *F. prausnitzii* abundance could promote a pro-inflammatory state similar to MetS (Koren *et al.* 2012, Garcia-Mantrana & Collado 2016, Nuriel-Ohayon *et al.* 2016, Ferrocino *et al.* 2018).

### Gut bacterial load and weight gain in pregnancy

Pregnant overweight women that harbour a high amount of energy harvesting microbes usually supply high amount of energy to the foetus. This could lead to a high birth weight and increased risk of complications to both the mother and newborn (Collado *et al.* 2008). The gut bacterial load increases throughout pregnancy irrespective of maternal body weight. Specifically, *Bacteroides. fragilis* and *Staphylococcus* have been shown to increase with weight gain as pregnancy advances in normal weight women (Collado *et al.* 2008, Santacruz *et al.* 2010). Furthermore, maternal pregestational BMI is inversely related to gestational weight gain. For example, obese women show decreased gestational weight gain (Broskey *et al.* 2017, Zacarías *et al.* 2018); and a possibly related decrease in *Bacteroides* by third trimester (Zacarías *et al.* 2018).

At 30–35 weeks of gestation, normal weight women showed higher *Bacteroides* and lower *Faecalibacterium* compared to overweight women (Zacarías *et al.* 2018). They also had lower *Coprococcus*, *Blautia*, *Catenibacterium* and *Actinomyces* compared to obese pregnant women at the same gestation (Zacarías *et al.* 2018). Normal pregestational BMI is associated with reduced *Firmicutes* – *Blautia*, *Coprococcus*, *Anaerostipes*, *Holdemenia*, and *Bulleidia*; and increased *Bacteroides*, *Coriobacteriaceae* (*Collinsella*, *Eggerthella*, *Atopobium*) and *Methanobrevibacter* at third trimester. High pregestational BMI (i.e. obesity) is associated with a high amount of *Lachnospiraceae*. These unique microbial compositions could partly explain the third trimester-associated elevated *Firmicutes*:*Bacteroidetes* ratio observed in overweight/obese women compared to their normal weight counterparts (Zacarías *et al.* 2018).

*B. fragilis* also has an LPS-dependent pathologic association with diabetes (Mancuso *et al.* 2005). *Methanobrevibacter* is known to enhance energy extraction by utilisation of H_2_ produced by bacterial fermentation of dietary fibres (Amabebe *et al.* 2020). *Coriobacteriaceae* adversely affect plasma cholesterol metabolism, that is, positively correlated with non-HDL-C (Clavel *et al.* 2014); and are enriched in obese animals (Clarke *et al.* 2012). Members of this family have been identified in cases of IBD, periodontitis, colon cancer, diabetes and bacteraemia. *Enterobacteriaceae* (*E. coli*) also increase with weight gain as gestation advances (Santacruz *et al.* 2010). These gestational weight-associated bacterial community alterations are usually associated with lower within subject α-diversity (Koren *et al.* 2012, Stanislawski *et al.* 2017, Zacarías *et al.* 2018), pro-inflammation, insulin resistance and hyperglycaemia (Qin *et al.* 2018).

In addition, *Bifidobacterium* and *Akkermansia muciniphila* decrease with weight gain over gestation (Collado *et al.* 2008, Santacruz *et al.* 2010). High bifidobacteria and *A. muciniphila* correlate with reduced inflammation, improved glucose tolerance and insulin sensitivity (Vyas *et al.* 2019, Amabebe *et al.* 2020, Xu *et al.* 2020). Therefore, low amounts of these butyrate-producing species as observed with increased maternal weight gain towards the third trimester may contribute to the inflammatory processes and MetS-like phenotype associated with this stage of pregnancy (Fig. 2) (Collado *et al.* 2008).

Changes in gut microbiota influence host gestational weight gain through increased glucose and fat absorption, enhanced secretion of fasting-induced adipocyte factor (FIAF), induction of catabolic pathways, and stimulation of a low-grade pro-inflammatory state (Bäckhed *et al.* 2007, Collado *et al.* 2008, Koren *et al.* 2012). Taking the studies of Collado *et al.* (2008), Santacruz *et al.* (2010), Koren *et al.* (2012) and Liu *et al.* (2017) together, the increased energy extraction and pro-inflammation observed in the third trimester of normal pregnancy may be the result of a synergy between *Proteobacteria* (*Enterobacteriacaea*), *Actinobacteria* (*Propionibacterium*), *Tenericutes* and some *Firmicutes* (non-butyrate producers). The inflammation is further promoted by the reduction in *Faecalibacterium*, *Bifidobacterium* and *A. muciniphila* and increase in obesogenic/diabetogenic *Bacteroidetes* such as *Prevotella* spp. and *B. fragilis* with resultant high circulating LPS levels and insulin resistance (Figs 1 and 2). This diabetogenically favourable gut microbial synergy at the later stage of gestation warrants further investigation especially at bacterial genus, species as well as metabolite levels. Besides, the type and level of metabolites produced by the bacteria may be the ultimate determinant of the observed inflammatory status.

Nevertheless, gut microbiota may also contribute to gestational dysmetabolism and inflammation through other yet unresolved mechanisms. Disease-associated correlations between specific taxa and gestational metabolic markers have been identified. For example, high amount of lactate-producing *Collinsella* (*Actinobacteria*) is associated with increased circulating levels of insulin, homeostatic model assessment of insulin resistance (HOMA-IR), triglycerides, and very-low-density lipoproteins. Similarly, *Sutterella* (*Proteobacteria*) and C-reactive protein (CRP) – a marker of chronic low-grade systemic inflammation as seen in obesity, diabetes, heart disease and colorectal cancer (Zacarías *et al.* 2018); *Ruminococcaceae*/*Lachnospiraceae* and leptin; *Bacteroidaceae* and ghrelin; *Coprococcus* and gastrointestinal polypeptide (GIP) are directly related. In addition, high abundance of *Blautia* spp. is associated with low insulin, HOMA-IR and HbA1c; increase in *Faecalibacterium* or *Faecalibacterium*/*Fusobacterium* ratios is associated with low fasting blood glucose; and high butyrate-producing *Odoribacter* (*Bacteroidetes*) is associated with low arterial blood pressure in overweight pregnant women. Similar inverse relationships have been reported for *Ruminococcaceae* and GIP, as well as *Prevotellaceae* and ghrelin (Ferrocino *et al.* 2018, Ponzo *et al.* 2019).

## Elevated production of pro-inflammatory mediators

In normal pregnancy, there is increase in body fat in early gestation and subsequent reduced insulin sensitivity (Barbour *et al.* 2007). Reduced insulin sensitivity correlates with elevated levels of circulating chemocytokines for example, TNF-α, IL-6 and IL-8 (Kirwan *et al.* 2002) that trigger obesity-associated metabolic inflammation (Gregor & Hotamisligil 2011). Though these inflammatory responses are detrimental to long-term health in the obese state, increased adiposity, hyperglycaemia and decreased insulin sensitivity favour foetal growth and prepare the mother’s body for the energetic demands of parturition and lactation. Therefore, the pro-inflammatory state is beneficial in the context of a normal pregnancy (Di Cianni *et al.* 2003, Lain & Catalano 2007, Nelson *et al.* 2010, Koren *et al.* 2012).

The increased abundance of gut *Proteobacteria* as pregnancy advances is associated with significantly higher levels of IFN-γ, IL-2, IL-6, and TNF-α in third trimester than in the first (Fig. 1) (Koren *et al.* 2012). The serum, adipose and placental tissue levels of these pro-inflammatory mediators are seen to increase later in pregnancy of humans with the mucosal surfaces of the entire gastrointestinal tract reflecting a low-grade inflammatory state (Cani *et al.* 2012). Transfer of gut microbiota from healthy women in third-trimester of pregnancy to a gem-free mice stimulated increased adiposity, insulin resistance and increased production of pro-inflammatory cytokines including IL-1β, IL-2, IL-5, IL-6 and GM-CSF compared to mice inoculated with microbiota from healthy women in the first trimester of pregnancy (Koren *et al.* 2012). This is somewhat akin to the impact observed when gut microbiota from non-pregnant obese human donors are transferred to gem-free mice (Ridaura *et al.* 2013). That this gestation-related pro-inflammatory and diabetogenic gut microbiota is beneficial to foetal development and prepares the mother for postnatal nurturing is thought provoking. Understanding the factors (genetic or otherwise) underlying these fetomaternal microbial, immune and metabolic interactions could guide diagnostic and therapeutic strategies for conditions such as gestational diabetes and pre-eclampsia.

Zacarías *et al.* (2018) have recently demonstrated another evidence in support of the association of pregnancy-related gut microbial changes and inflammation. Similar to obesity (Turnbaugh *et al.* 2009, Fu *et al.* 2015), third trimester gut microbiota showed lower α-diversity (similar to Koren *et al.* 2012) that was associated with increased circulating high-sensitive CRP (hs_CRP) and haptoglobin – both confirmed markers of inflammation characteristic of metabolic diseases such as obesity, diabetes, cancers, etc. Reduced gut microbial diversity and richness is associated with increased adiposity and inflammatory profile (Cotillard *et al.* 2013, Verdam *et al.* 2013). In general, serum levels of both inflammatory mediators increased with high amounts of *Firmicutes* – *Blautia*, *Coprococcus*, *Anaerostipes*, *Holdemenia*, and *Bulleidia*; and reduced amounts of *Bacteroides*, *Coriobacteriaceae* and *Methanobrevibacter*. Individually, hs_CRP was inversely related to *Faecalibacterium*. Haptoglobin which also acts as a chemoattractant for macrophages in adipose tissue increased with low *Firmicutes* (normal weight women) and low *Bacteroidetes* (overweight women). Haptoglobin was also directly related to *Firmicutes* in overweight women; and generally to *Ruminococcus gnavus* (a member of *Clostridium* cluster XIVa – mucosal butyrate producers) (Zacarías *et al.* 2018). The mechanisms underpinning these observations and subsequent clinical implications are poorly understood. Whether they are exclusively related to the beneficial diabetogenic state of third trimester needs to be determined by further *in vivo* and *in vitro* experiments.

## Effect of oestrogen and progesterone on gut function

Some researchers have hypothesised that high levels of pregnancy-related hormones that increase gut transit time could be an adaptation that facilitates enhanced nutrient and energy harvest, thereby promoting weight gain in pregnancy (Edwards *et al.* 2017). This is based on the effect of the significant rise in ovarian hormones during prenatal and postpartum periods on intestinal contractility and transit (Mulak *et al.* 2014). Physiologic hyperoestrogenaemia and hyperprogesteronaemia as seen in later stages of pregnancy can reduce gastrointestinal contractility and prolong transit (Fig. 1) (Mulak *et al.* 2014). This can encourage enhanced nutrient and energy extraction especially in the abundance of energy harvesting microbes such as *Tenericutes* and some *Firmicutes*. These symptoms are similarly observed in the context of irritable bowel syndrome (IBS) (Mulak *et al.* 2014).

Furthermore, oestrogen and progesterone can directly modulate gut microbiota composition, bacterial metabolism, growth and expression of virulence factors (Mulak *et al.* 2014). It appears the ovarian hormones participate in creating a suitable environment for gut microbial-nutrient interaction that leads to increased nutrient digestion and absorption (Fig. 1). This manifests as weight gain for both mother and foetus and a MetS-like phenotype.

Ovarian hormones also influence insulin sensitivity during pregnancy (Bruns & Kemnitz 2004). Maternal insulin resistance is associated with increasing levels of progesterone and oestrogen in the third trimester. This supports rapid foetal growth by promoting the preferential transfer of carbohydrates to the foetus (Buchanan 1991). The foetus also produces high amounts of insulin in response to excess glucose, and foetal insulin stimulates growth *in utero* (Bruns & Kemnitz 2004). Some mothers are unable to compensate adequately for this hormone-induced insulin insensitivity, and gestational diabetes ensues (Buchanan 1991). On the other hand, in a vast majority of pregnant women, a compensatory mechanism(s) ensures a reversal to normal insulin sensitivity and glucose tolerance indicating a beneficial effect of the hormone-related diabetogenic phenotype. Women who develop gestational diabetes are unable to produce an adequate amount of insulin to maintain euglycaemia, and exhibit greater insulin resistance with resultant glucose intolerance (Hod *et al.* 2015).

## Microbiota-metabolite profile

Another plausible mechanistic pathway to the beneficial diabetogenic phenotype characteristic of the later stage of gestation is the microbial-induced disequilibrium in production of SCFAs – acetate, butyrate and propionate (Fig. 2). SCFAs are derived from intestinal bacterial fermentation of indigestible dietary fibres. They are energy substrates and regulate satiety and food intake. They regulate glucose and energy homeostasis and metabolism through both local (intestinal) and systemic (brain, liver, adipocytes, skeletal muscle etc.) effects. They also enhance intestinal epithelial barrier integrity, and inhibit bacterial translocation and inflammation. The roles of SCFAs in control of body weight, obesity and diabetes in no*n*-pregnant state is well defined (Canfora *et al.* 2015, Saad *et al.* 2016, Vyas *et al.* 2019, Amabebe & Anumba 2020, Amabebe *et al.* 2020).

Butyrate produces more potent anti-inflammatory responses compared to acetate and propionate (Cox *et al.* 2009, Mirmonsef *et al.* 2011, 2012, Koh *et al.* 2016, Rivière *et al.* 2016, Parada Venegas *et al.* 2019). The dysbiotic microbiota at late gestation displays a local dysmetabolism characterised by reduced butyrate levels relative to acetate, propionate and lactate. In conjunction with increased circulating LPS from the Gram-negative bacterial species (e.g. *Bacteroides*, *Prevotella* and *Enterobacteriaceae*), decreased butyrate could create an immune imbalance in favour of a pro-inflammatory state with an elaborate systemic dysmetabolism – insulin resistance, hyperglycaemia and increased weight gain, albeit not injurious in this context (Fig. 2).

The declining butyrate level might be the determining factor that perpetuates the pro-inflammatory state, as this state is maintained despite an increase in acetate, propionate and lactate that are somewhat anti-inflammatory. For example, *Proteobacteria* with adherent-invasive capacities (*E. coli*, *Campylobacter concisus*, and enterohepatic *Helicobacter*) are active components of IBD (Mukhopadhya *et al.* 2012); and *E. coli* colonisation alone induce sufficient macrophage infiltration of white adipose tissue, reduced insulin sensitivity and glucose intolerance in germ-free mice (Caesar *et al.* 2012). Butyrate has found extensive therapeutic use against IBD due to its potent anti-inflammatory actions compared to the other SCFAs (Silva *et al.* 2018, Chen & Vitetta 2020). Chen and Vitetta (2020) have recently highlighted the clinical utility of the extensive anti-inflammatory properties of butyrate and advocated butyrate supplementation (singly or in combination with other anti-inflammatory agents) as a practical approach in treating IBD. These evidence highlight the importance of butyrate in the inflammation equation linked to gestation-induced gut dysbiosis. These hypotheses are yet to be explored in relation to the physiology of pregnancy and parturition. However, longitudinal metabolomics of the gut microbiome in pregnant women and postnatally could provide more insight on the benign effect of this dysbiotic, dysmetabolic and inflammatory phenotype, instead of an outright disease state.

## Conclusion and future perspectives

Despite its devastating clinical appearance, the prognosis of MetS-related features may not necessarily be deleterious to the host or foetus in the case of pregnancy. During normal pregnancy, the disease-associated gut microbial dysbiosis and related immunological responses that increase the risk of obesity and diabetes create an interestingly favourable metabolic environment that preferentially supplies sufficient nutrients for rapid foetal growth and development while preparing the mother for parturition and lactation. At third trimester, the gut microbiota-induced energy metabolism that encourages energy efficacy and storage (Garcia-Mantrana & Collado 2016) shifts in favour of the foetus who requires an adequate supply of nutrients for rapid growth. Concomitantly, a transient maternal reduced insulin sensitivity creates a benign diversion of nutrients for foetal use instead. Similar to obesity, the insulin resistance is triggered by a low-grade systemic inflammatory state. The increased weight gain (increased fat and energy storage) in the mother supplies the energy required for delivery and lactation.

In some cases, these benign processes can become pathologic leading to gestational diabetes and pre-eclampsia. However, a potential overt inflammation and dysmetabolism is usually averted by yet unresolved compensatory mechanisms. The gut microbiota associated with this beneficial diabetogenic phenotype at late gestation is believed to be characterised by an increase in energy harvesting microbes that are usually associated with obesity and diabetes in non-pregnant state, that is, increase in genera/species of *Enterobacterioceae*, *Propionibacterium*, *Tenericutes*, and an increased *Firmicutes*:*Bacteroidetes* ratio; but low in butyrogenic bacteria – *F. praustnitzii*, *A. muciniphila* and *Bifidobacterium* (Collado *et al.* 2008, Santacruz *et al.* 2010, Koren *et al.* 2012, Liu *et al.* 2017). *Proteobacteria* (*Enterobacterioceae*) is even suggested to be a predictive biomarker of diabetes (Amar *et al.* 2011). The increase in *Bacteroides* spp. and accompanying high LPS levels are also important processes that contribute to the inflammation.

Generally, the non-butyrate producers, which are coincidentally high LPS bearers, appear to be the proponents of this benign dysbiosis-associated pro-inflammatory state. Perhaps high LPS and low butyrate constitute the recipe that triggers this gestation-related benign diabetogenic state. Some authors suggest that maternal BMI prior to pregnancy is linked to the dysbiotic gut microbiota and inflammatory status at third trimester (Zacarías *et al.* 2018).

Of course, a dysbiotic gut microbiota deficient in butyrogenic bacteria and enriched with LPS bearers is associated with obesity and its comorbidities, which increase an individual’s risk of pre-eclampsia and gestational diabetes. However, a similar microbial-metabolic profile commonly observed in the later stage of pregnancy does not produce the same harmful prognosis. The associated microbial composition and physiologic mechanistic pathways may be influenced by yet undetermined genetic and environmental factors. Additionally, genus and species level resolution of the hormone and inflammation-induced microbial changes could validate these observations and improve our understanding of the mechanisms involved. It may also elucidate the microbial, hormonal and/or immunologic ‘threshold’ for transition from the beneficial physiologic metabolic phenotype to pathologic states such as gestational diabetes, pre-eclampsia, etc.

In conclusion, though the gut microbiota influence host energy metabolism and homeostasis, and a dysbiosis is usually injurious to the host, the overall outcome of changes in gut microbiota structure and composition varies depending on the pregnancy status of the individual. The altered gut microbiota that connotes disease state in non-pregnant individual, promotes metabolic and immunological changes beneficial to the fetomaternal unit and success of pregnancy.

## Declaration of interest

The authors declare that there is no conflict of interest that could be perceived as prejudicing the impartiality of this review.

## Funding

Though this research did not receive any specific grant from any funding agency in the public, commercial or not-for-profit sector, E A and D A are funded by National Institute for Health Research http://dx.doi.org/10.13039/100005622 (NIHR, 17/63/26).

## Author contribution statement

Conceptualization and literature search – E A. Original draft preparation, review and editing – E A and D A. Both authors approved the final version of the manuscript for submission.
